# A bibliometric analysis and literature review on emotional skills

**DOI:** 10.3389/fpsyg.2023.1040110

**Published:** 2023-05-24

**Authors:** María Teresa Manjarres, Diana Paola Mahecha Duarte, Jorge Navarro-Obeid, Maria Laura Vergara Álvarez, Isneila Martinez, Lorena Cudris-Torres, Juan Hernández-Lalinde, Valmore Bermúdez

**Affiliations:** ^1^Universidad Nacional abierta y a distancia, Corozal, Sucre, Colombia; ^2^Corporación Universitaria del Caribe, Sincelejo, Colombia; ^3^Fundación Universitaria del Área Andina, Bogotá, Cundinamarca, Colombia; ^4^Universidad Simón Bolívar, Cúcuta, Colombia; ^5^Centro de Investigaciones en Ciencias de la Vida, Universidad Simón Bolívar, Barranquilla, Colombia

**Keywords:** Well-being, mental health, personality, bibliometric analysis, bibliometrix

## Abstract

The content, management, and implementation of social skills have been developed since the end of the 20th century as a model of capabilities. Thus, as human beings develop and train their basic cognitive and perceptual–motor functions, they increase their ability to solve and cope with difficulties. This article aims to present a bibliometric and systematic review of social skills, using query sources in databases such as Web of Science (WoS) and Scopus between the years 2000 and 2022, with platforms such as Bibliometrix and Gephi. This search yielded a total of 233 records in WoS and 250 records in Scopus that were merged and, after eliminating 143 duplicate data, were consolidated into 340 records that enclose the academic production of 20 years. Through scientific mapping, the main authors, journals, and countries in this field were determined; similarly, the most relevant studies were classified into three categories, namely, classic, structural, and perspectives, which were represented by means of the metaphor of the tree of science. In addition, a program for further studies was planned, such as in-depth qualitative research measuring observationally and directly taking into account emotional expressiveness, emotional understanding, emotion regulation, and behavior, and the impact of social skills training on social problem-solving. Finally, another important aspect to mention is that this research work is useful for the scientific academic community in many areas of knowledge such as psychology, education, and managers of educational institutions.

## 1. Introduction

Globally, the coronavirus disease 2019 (COVID-19) pandemic has influenced emotions (Mangiavacchi et al., 2021) affecting people’s quality of life because of blockages, lockdowns, physical distancing, restriction of social interactions, deprivation of traditional learning methods, and situations that have caused stress, anxiety, and mental health concerns (Salmela-Aro et al., 2021), that is, it is increasingly difficult to ignore emotional situations that affect mental health in human beings (Lane and Smith, 2021).

In this sense, psychosocial problems have become one of the concerns of daily life, especially for those people who perform activities of direct contact and care, mainly in the educational field (Schoeps et al., 2019). As it has been observed, educational requirements demand productivity, warmth, attention, quality, and even conflict resolution, the latter having the capacity to solve unexpected situations (Peláez-Fernández et al., 2021). To this end, the scientific literature recognizes emotional skills (ES) as a fundamental axis for managing emotions, feelings, assertiveness, empowerment, and solving difficult situations (Aly et al., 2021). This is how managing social skills is closely related to the emotional ability that human beings show for the development of emotions (Estrada et al., 2016).

In this regard, some reviews were found that relate, for example, a meta-analysis exploring psychoeducation and the component training of cognitive emotional skills development in children and adolescents (De Mooij et al., 2020); a literature review that proposes the development of tools and training based on simulation and measure design for decision-making (Stalnikowicz and Brezis, 2020); a review that proves the benefits of video game training on cognitive and emotional skills in relation to healthy young adults (Pallavicini et al., 2018); another meta-analysis that explores the key individual and environmental factors, related to identity and social–emotional skills (SS) such as continuous reciprocal interactions between the self and peer environment, emotion regulation, and social skills for general wellbeing and adequate positive mental health (Mitic et al., 2021); and finally, a systematic review that proposes organizing relevant concepts of alexithymia or the inability to identify one’s emotions and consequently, an inability to express what clients are feeling to help therapists to get a clear idea of how clients function and why they transmit feelings the way they do (Nunes da Silva, 2021).

On the other hand, emotional competencies are conceived from childhood, forging a result of free and own learning strategies, which are permanent and teach the subject to create emotional stability, self-esteem confidence in their capabilities, good decision-making, and skills to relate with others; in short, strategies to be a happy individual in society (Gallego-Tavera et al., 2021), which allows its applicability to be used in different contexts of human being’s performance forming ideal welfare through the balance of emotional, mental, and social factors. Thus, educational institutions are positioned as the entities with the greatest advancement of emotional skills and where the factors and constituent elements of emotional intelligence should be applied the most (Cohen and Syme, 2013). Without leaving aside, the family constitutes a fundamental part of support to the institutions since the family favors with the communication and development of school activities (Pérez-Fuentes et al., 2019).

The strengthening of these competencies is a great challenge for society, especially in relation to the adaptation and performance of the subjects in each of their environments. Thus, it is necessary to strengthen skills focused on self-regulation and control of emotions. The above is a strategy that contributes to minimizing the emotional burden faced by the subjects nowadays as a result of relevant aspects such as inequalities, inequities, and even more the aftermath generated by the Pandemic. In view of the aforementioned, situations evidenced in intolerance to frustration and limited capacities to provide solutions to the problems presented are highlighted, evidencing deficiencies in the appropriation of psychological resources, especially those related to emotional skills (Martinez et al., 2023).

In relation to the above, in the 1990s, UNESCO presented a report on education for all, focusing on the need for lifelong learning, based on the integral development of subjects and the improvement of their quality of life, informed decision-making, and centered on education for all. At the same time, the World Health Organization promotes an initiative called life skills, focusing on the consideration of social skills, empathy, management of emotions and feelings, stress and tension management, and prevention of psychosocial risks. Similarly, the Organization for Economic Cooperation and Development (OECD) from its lines of research emphasizes the study of social and emotional skills and how they can become predictors of success. In its report on Competencies for Social Progress, it states that the school population requires a balanced endowment of cognitive, social, and emotional skills to achieve positive life outcomes, and, therefore, the implementation of public policies aimed at this purpose is essential (Organization for Economic Co-operation and Development (OECD), 2015). Currently, from the World Education Summit, a reference is made to determine elements, such as solidarity and cooperation, to contribute to collective work and empathy.

The subject of interest is visible in various areas of human interaction, such as in the school environment, a predictor of success is academic performance, which over time has been related to intellectuality, but the presence of skills that promote the capacity for self-regulation, as well as decision-making and problem-solving, and aspects that contribute to psychological well-being and healthy lifestyles, should not be overlooked. In addition, these types of positive experiences contribute to the wellbeing of teachers and students, generating favorable school spaces (Gordón et al., 2019). Without leaving aside, the family environment, considered a protective environment, can provide individuals with the necessary tools to face different situations.

On the other hand, from the professional point of view, psychosocial conflicts should be the object of interest, especially those that promote interaction and direct contact with different population groups. It should be noted that in this context, the occurrence of stressful events is a recurrence that invites the generation of coping techniques and control of emotions (Laudadío and Mazzitelli, 2019).

In the interpersonal sphere, emotional skills are evident, due to the great significance of empathy, and the ability to understand other emotional states, as well as the ability to cope with various situations. In addition, this is considered a predictor of job success (Galindo, 2018).

Studying the development of emotional skills is of great interest due to the implications present in the daily life of the subjects, especially in the school population, since it contributes to the regulation, control, and recognition of these skills (Merchán-Clavellino et al., 2019). It should be noted that emotional skills become predictors of functioning, so those who are considered emotionally intelligent have strengthened the ability to perceive, understand, and manage emotions.

Other relevant aspects as mentioned by Lucero (2015) are the research contributions, from a cognitive perspective where it has been possible to relate the existing relationships between failure and success from various factors such as social perception, cognitive styles used to respond to the various problems, and the coping of each situation at different stages of the life cycle. Another significant aspect is the integration of emotional variables associated with protective factors related to mental health and vulnerability, as well as the effects on it. In relation to the above, it is established that positive emotions have the possibility of identifying negative effects resulting from poverty experiences, acting as protective factors (Seligman Campayo Muñoz and Cabedo Mas, 2017).

Therefore, emotional competencies can be understood as the set of skills possessed by human beings to understand, comprehend, develop, accept, and adapt individually and socially in society, achieving understanding with their peers.

In this order of ideas, the main objective of this article is to develop a bibliometric and systematic review of SE, to answer the following research questions: Which authors, countries, and journals lead the literature on emotional skills, and which are the most-cited authors? What are the main research papers, the main areas, and what perspectives or trends exist in the literature on this field of study? This is necessary to achieve an approach that shows the importance and benefits in the social context of the implementation and continuous training of social skills in human beings in terms of mental health and preservation of life, which are supported by a chronological vision that shows the variables presented by research professionals, universities, and other entities.

This article is organized as follows: The first part presents the introduction of the study, including a contextualization of the problem, the importance of the topic, and literature review on emotional skills, the main objective, and the research questions. The second part shows, in detail, the methodology developed in two phases: a scientific mapping of the area through a bibliometric analysis and, the second phase, a network analysis to identify the most relevant papers on social skills and to determine the main groups on which current research is focused. The third part presents the results with their respective discussion. Finally, the conclusion and recommendations for future research are presented.

## 2. Materials and methods

This review is developed in two phases—the first with a scientific mapping of the area through a bibliometric analysis of the scientific production registered in Scopus and WoS in a time range between 2000 and 2022; this is due to the great relevance of this tool in current bibliometric studies that are allowing the recognition and development of trends in thematic areas and their basic structures (Bonilla-Chaves and Palos-Sánchez, 2023). Similarly, other recent research has used bibliometric analysis by means of the Bibliometrix application to perform exhaustive and comprehensive analyses of the scientific mapping of a specific topic (Lizano-Mora et al., 2021; Palos-Sanchez et al., 2022). However, for the present research, as selection criteria for the articles, a search was conducted in English to obtain the largest number of documents in the WoS and Scopus databases from the year 2000 to the first quarter of the year 2022. As search criteria, the title, the scientific journals indexed in Scopus and WoS, and the search term “emotional skill” were taken into consideration. The second phase with a network analysis allows identifying the most relevant papers on social skills and determines the main clusters on which current research on the area is focused (Cristóvão et al., 2017; Rojas-Sánchez et al., 2022; Park and Jeon, 2023).

### 2.1. First phase: scientific mapping

To perform a production analysis and scientific mapping, the five bibliometric methods suggested by Zupic and Čater (2015) were used: citation analysis, word co-occurrence analysis, co-citation analysis, co-author analysis, and bibliographic coupling analysis. They were used jointly in WoS and Scopus since it allows a broader overview of the area of knowledge (Echchakoui, 2020). In order to clean the data, we filtered both in the Scopus database and in Web of Science, according to the search criteria, as shown in Supplementary Table 1. The results obtained were merged and duplicates removed, using the R estudio software, i.e. uniform articles found in the two databases were excluded. The search parameters are given in Supplementary Table 1.

These search criteria showed 233 records in WoS and 250 records in Scopus that were merged and, after eliminating 143 duplicate data, were consolidated into 340 records which enclose the academic production for 20 years (see Supplementary Figure 1). The estimated studies were published in specialized journals in the field of science area; an overlap of 14% was observed between these two databases, which demonstrates the relevance of using them together.

While reviewing in the search parameters the concept of “emotional skill” in different languages (French, English, German, and Spanish), it is intended to cover the largest number of records within these databases. As a result, it is identified that 77% of the publications in this area related to WoS and Scopus are in English, while 14% are in Spanish and 4% are in German (Supplementary Figure 2). This is because English is the dominant language in these databases; thus, these journals and authors attempt to perform their publications in English to increase their visibility (Vera et al., 2019). The tool used for the bibliometric analysis is Bibliometrix (Aria and Cuccurullo, 2017) since it is a free-use tool that allows working with different databases and multiple functionalities; it has also been used and validated by other studies (Acevedo et al., 2020; Duque et al., 2020, 2021c; Di Vaio et al., 2021; Queiroz and Fosso Wamba, 2021; Secinaro et al., 2021).

### 2.2. Second phase: network analysis

The research obtained in WoS and Scopus were grouped, and the duplicates were eliminated using the programming in the R Software. Their references were then extracted, and a citation network was structured using graph theory as a model, which is a tool that allows organizing information on the typology and characteristics of the network and all the studies that constitute it (Wallis, 2007; Yang et al., 2016).

Subsequently, three bibliometric indicators were calculated: the indegree indicator (number of times in digits that a study has been referenced by others; Wallis, 2007), the outdegree indicator (number of times in digits that a particular group cites others or the number of connections of each study; Wallis, 2007), and the betweenness indicator (degree of intermediation and centrality of each element within the network; Freeman, 1977). The latter is presented when the document is referenced and reference to the others (Zhang and Luo, 2017).

The result shows the knowledge structure in this area, organized by all the research obtained from the databases and their appropriable references, which allows work from various sources to be involved, not only those that are part of WoS and Scopus but also from other databases and scientific publications. This network analysis, also known as co-citation mapping, helps visualize the structure of an area of knowledge and also facilitates identifying its subareas or research tendencies (Gurzki and Woisetschläger, 2017; Zuschke, 2020). To provide graphical visualization of the knowledge network of the emotional skills study area, the tool Gephi is used (Bastian et al., 2009).

The indegree, outdegree, and betweenness indicators were calculated for each record of the network, which allows classifying the works using the tree metaphor (Robledo et al., 2014; Valencia et al., 2020). Implementing this analogy, three categories arise as follows: the roots (high indegree), where classical documents and documents of theoretical hegemonic relevance within the field of study are identified, especially publications that are cited but do not cite others (Wallis, 2007); consecutively, in the trunk (high betweenness), those documents that cite but at the same time are cited by others are found (Zhang and Luo, 2017). In this topic, structural works are grouped that link the theoretical foundation of the classics with current research; and finally, the leaves (high outdegree), where most of the recent studies are found and others are cited (Wallis, 2007); these publications show the current framework guidelines for research in the area that are established as emerging research fronts. This methodological process has been used and validated in previous studies (Clavijo-Tapia et al., 2021; Ramos et al., 2021; Torres et al., 2021; Trejos-Salazar et al., 2021; Duque et al., 2021a,b; Rubaceti et al., 2022).

## 3. Results

### 3.1. Bibliometric analysis development

A bibliometric analysis of the scientific production recorded in WoS and Scopus was performed. Supplementary Figure 3 shows the number of publications between 2000 and 2022. In total, 340 records related to ES were identified. A growing trend in the number of records was visualized, showing an annual growth rate of 15%. Furthermore, it was visualized that more than half of the publications were made in the last 5 years. These data show an increase in the interest of the scientific community in the subject during the last decade.

Supplementary Table 2 shows the ten countries that provide the largest number of publications. The United States is identified as the country that leads the list with 16.1% of the total number of publications, followed by Spain with 11.1%, and the United Kingdom with 4.7%. In general terms, of the 10 countries with the largest publications, seven are European and contribute 47.7% to the field of study, representing the importance of this continent in the study, benefits, contributions, and search for integral wellbeing of social skills. The American continent represented by the United States and Mexico represents 18% and Oceania with a unit represented 3.5% of publications.

Supplementary Table 3 shows the ten authors with the highest number of publications, the number of citations, and their corresponding index *h*. Filip K. Fruyt ranked first from the Universiteit Gent, Ghent (Belgium); however, the author with the highest number of citations is Oliver P. John who, in turn, is the author with the highest index *h*.

Supplementary Table 4 lists the scientific journals with the largest number of associated articles. In addition, the index h and the quartile in which they are found are presented. Only one of the journals is in Q1, four are in Q2, and three do not record quartiles. The journal with the most publications contributing to the subject of study is the Frontiers of Switzerland with a total of eight records, as well as Index *h* and the highest SJR.

Supplementary Figure 4 shows the four main components that make up the bibliographic analysis. In the first square is the author co-citation network, which made it possible to identify the most prominent authors in terms of citation count; in this study, Joseph A. Durlak, John D. Mayer, and Peter Salovey are the most referenced. The second square shows the network of authors’ collaboration and shows the joint work of the authors who had already been mentioned above, such as Filip K. Fruyt, John Oliver, Ricardo Primi, Daniel Santos, and their close literary link. The collaboration network between countries reaffirms the leading role of the United States, Belgium, and Brazil. Finally, the term co-occurrence network is given, where two large groups of compound words are found: the first (in red) by words such as emotions, human beings, children, and adults, and the second (in blue) illustrating variables such as adolescents, emotional intelligence, empathy, and skills, the latter being constant in both groups.

### 3.2. Network analysis

Through this analysis, it was possible to identify the most important documents in the area. Documents with the highest indicators were chosen for review and were organized using the science tree metaphor—five classics (roots), five structural (trunk), and 10 leaves (leaves). To establish the subareas or common areas of research, the clustering algorithm proposed by Blondel et al. (2008) was used. In this way, three main groups were identified that can be represented in the leaves (Supplementary Figure 4).

#### 3.2.1. Root (classics)

The hegemonic publications (tree roots) that make up this literature review are considered research that supports the study subject. These documents generally raise the importance and assessment of competencies in ES for children, youth, and parents.

One of the most referenced documents, considered a seminal work in the area, is “emotional intelligence” by Salovey and Mayer (1990), who determined that mental processes and individual differences are related to emotions, and their use and regulation in an adaptive way. Subsequently, Goodman (1997) offers a brief evaluation of behavior, strengths, and difficulties through the qualities and difficulties questionnaire (SDQ) for children and youth and Rutter’s questionnaires for parents, which allow evaluating and showing a simple report of emotional and behavioral difficulties, problems with peers, hyperactivity, and prosocial behaviors.

Furthermore, Durlak et al. (2011) explored the effects of social–emotional learning (SEL) programming on social and emotional skills, attitudes toward self, positive social behavior, behavioral problems, emotional distress, and academic performance in different schools in the United States. Similarly, years later, Taylor et al. (2017) promoted personal strengths in young people, social competencies, positive identity, and commitment to learning, classifying the results into seven categories that evaluated positive social and emotional assets (SS and ES and attitudes toward oneself, others, and school) and wellbeing indicators—positive (positive social behaviors and academic performance) and negative (behavior problems, emotional distress, and substance use).

Finally, Cetina et al. (2011) highlight that parents who take into consideration the dimensions of emotional warmth and train themselves in ES promote self-regulation in children, greater self-esteem, and a greater adjustment in psychological maturity.

#### 3.2.2. Trunk (structural)

This section identifies the publications indicating the research that links what is shown by the authors and the documents of classical foundation with the most recent authors and approaches on ES, being a category that shows the models and orientations consolidating the scientific study in this field.

Delhaye et al. (2013) described attachment and self-report measures in SS considering emotional intelligence (EI), empathy, and resilience. Moreover, Björklund et al. (2014) showed that models based on SEL allowed enhancing children’s social interaction skills and emotion management to contribute to promoting mental health. Similarly, Appelqvist et al. (2016) implemented and evaluated a method to improve SS and prevent psychosocial problems in children between 7 and 12 years, expressing that children with low social ES are at a greater risk of connecting with school and having good social relationships in the future.

Moving on with structural research, Schoeps et al. (2018) proposed that learning ES is a potential component to promote the quality of interpersonal relationships, wellbeing, and emotional education in adolescents. In this line, Abrahams et al. (2019) analyzed how the measurement of SS in young people can advance in terms of conceptualization and classification of skills, techniques, and evaluation methodologies to improve wellbeing and subsequent positive results in life.

#### 3.2.3. Recent documents (leaves)

With the bibliometric review process, three main subareas (clusters) were established in this field of study which shows more recent lines of research. Each of them has been set out in the following section.

##### 3.2.3.1. Cluster 1: emotional skills and education

This perspective reflects the connection and benefits that ES present as an essential complement in education (Supplementary Figure 5), identifying studies such as that of Denham (2006) who suggests deepening the social–emotional domain in multiple contexts. Moreover, French and Mantzicopoulos (2007) applied the structure of the pictorial scale of competence and social acceptance (PSPCSA) to evaluate skills related to cognitive competence, acceptance by the mother, and acceptance by peers, finding a significant decrease in perceptions of cognitive competence; however, they concluded that it is not recommended that professionals only use the PSPCSA to make decisions about children. There is always the need to carry out an exhaustive study to know the evolution of children’s own beliefs. Similarly, Bierman et al. (2008) promoted the abilities of teachers to use research-based practices based on the development of SS through training workshops and tutorials with an intervention program.

Later, LeBuffe et al. (2013) assessed social and emotional competencies in children, using the Devereux set of assessments to measure protective factors related to resilience and believing that developing a child’s protective factors will mitigate the impact of risk. Subsequently, with a similar study, Denham et al. (2014) evaluated social aspects, self-regulation, problem-solving, and social–emotional behavior through SEL programming and the components of social learning theory, suggesting that early assessment and control are possible, using these measurement instruments to maximize early school success. Furthermore, McCoy et al. (2016) analyzed the progress of cognitive and socio-emotional skills in the early stages of life, concluding that countries that are exposed to risk factors, such as infectious diseases, malnutrition, poverty, and low availability of educational and healthcare resources, affect the low development of cognitive and socio-emotional skills of the child population.

Carrying on with the strategy of science tree reading, this perspective is finalized showing the last advances of Bethell et al. (2017) with a field study on promoting resilience, enhancing upbringing, and the social and emotional roots in childhood development, such as healthy components, promoting health for a lifetime, concluding that there are sufficient scientific findings that endorse building programs and policies applied to child health services from which four general priorities arose. First, resilience and enriching relationships in child health services; second, to cultivate conditions for cross-sectional collaboration to stimulate action and to approach structural inequalities; third, to recover and promote safe, enriching relationships and absolute participation of people, families, and communities to heal trauma; and finally, boosting efforts for research, innovation, and implementation of child and family health. At the same time, Wolf et al. (2017), through the international development and early learning assessment (IDELA), managed to evaluate the socio-emotional and motor needs of the child population to offer an integral education. It is also argued by a study that socio-emotional adjustment and children’s self-regulation is the key to the success and integral wellbeing of children (Vitiello et al., 2022).

##### 3.2.3.2. Cluster 2: emotional skills and personality

This perspective focuses on the effect of ES on the development of personality (Supplementary Figure 6). The most relevant research is the one raised by Primi et al. (2018) in which they analyzed socio-emotional skills related to the personality dimensions of Goldberg. Following this logic in another study, several conceptualizations of ES were examined, showing a relation between EI and the personality dimensions to raise an integrating set of domains of socio-emotional skills (Abrahams et al., 2019).

Currently, other research was identified that shows that social and emotional learning is fundamental to help in the decision-making of those in charge of public policies, to favor teachers in daily practice, and allow children and adolescents to reach their potential (Suarez-Alvarez et al., 2020). Primi et al. (2021), by means of the computer assessment of social and emotional skills using the child and adolescent inventory system (SENNA), demonstrated in their results that knowledge and understanding of specific skills are a high priority for handling multiplicity in self-efficiency, stability, self-management, creativity, and innovation.

In this order of ideas, Cieciuch and Strus (2021) highlight in the current knowledge a comprehensive model of flexible core personality competencies determined by stable temperamental traits with a biological base that underlies many specific emotional skills. Finally, Bhaktha and Lechner (2021) presented a simulation study regarding the performance of test scores and plausible values, in regression with scales of personality or socio-emotional skills as a forecast for school performance results and professional success.

##### 3.2.3.3. Cluster 3: emotional skills and autism

Under this perspective of ES and children with autistic spectrum disorder (ASD), in the first instance, Azevedo (2018) designed a digital game to promote the development of ES in children with ASD. The game considered rehearsed facial expressions of joy, sadness, fear, anger, and surprise in three different activities: impression, recognition, and narration. Sofronoff et al. (2017) examined an intervention in ES with the participation of parents of children with ASD, demonstrating significant improvements in children’s social skills, parents’ self-efficiency, children’s behavior, and anxiety levels.

Similarly, from the empirical studies performed, it is evident that there is a relation between how experiences mediated by music when learning languages can develop students’ interpersonal and collaborative skills to become active members of a more inclusive society (Cores-Bilbao et al., 2019). This situation leads Russo-Ponsaran et al. (2019) to demonstrate the usefulness, trustworthiness, and validity of the SEL web program concluding its usefulness and accessibility as a tool to measure complex profiles of socio-emotional skills in youth with ASD.

Finally, Hu and Lee (2020) evaluated the effects of cognitive-behavioral therapy in children with ASD with an ideal multiple training when it comes to acquiring and maintaining ES of acknowledging emotions in context, expression, seeking help, and regulation. Kastner et al. (2020) performed experimental research on educational programs in visual arts based on psychological theories and models of ES on how to teach, practice, and reflect skills or specific competencies to improve them through drawing portraits and focusing on instruction.

## 4. Discussion

The results of this research were presented through a generalized and quantitative analysis according to the search parameters on HE, with tables, graphs, and word clouds, emphasizing the main indicators of production and citation, continuing with an inductive type analysis that found three major groups in the cited articles, which are HE and education, HE and personality, and HE autism in children, youth, and adults.

At a general level, it is highlighted that the development and training of HE from childhood onward facilitate fundamental tools for conflict resolution, allowing them to interact peacefully and resiliently with others and achieve goals, is the result of favorable gains that allow human beings to acquire and strengthen many skills. HE training leads to open communication between individuals, allows for a better understanding of feelings, capacities and strengths, promotes them and enables people to develop confidence in themselves, in their abilities, to think assertively about problems, and to engage in conflict avoidance and resolution.

The above is consistent with Stalnikowicz and Brezis (2020), who suggest that skills such as empathy and listening are key challenges when communicating, affirming that emotional skills are required for the generation of trust and for the care of people’s health. Likewise, a meta-analysis similar to the present study explored individual and environmental factors associated with socio-emotional skills such as ongoing reciprocal interactions between self and peer environment, emotion control, and social skills as items of great importance for overall wellbeing and good positive mental health (Mitic et al., 2021).

Here lies the added value of this literature review because it highlights that the use and training of HE in an ideal way are a must in human beings, it is a shared responsibility, offering a base of studies at a global level allows foreseeing that there is a general interest in contributing to humanity for a better social coexistence that allows preserving mental health because it is made up of a balance of skills and positive and negative emotions throughout the development of the life cycle.

Thus, the HE and education scenario is highlighted with studies that give strength to the findings of this review, there is some research in this line that states that curricular studies on emotional skills make direct comparisons difficult, but suggest the importance and effectiveness of emotional skills in training for others. Equivalent to this, Campayo Muñoz and Cabedo Mas (2017) show that the commitment to emotional skills has benefits for the development of certain aspects and positive implications for education. Therefore, an adequate understanding of the relationship between aggressive behaviors and the level of HE in people would help the design of prevention programs and could improve intervention policies in this area of knowledge (Sylva, 2019).

In this order of ideas, the programs and all the trends mentioned are comprised of a set of instructional activities with goals and objectives designed to generate changes in attitudes and behaviors toward the resolution of interpersonal conflicts. They are fundamental to put them into practice and essentially from the school environment to leave a mark and to be able to continue training as the individual interacts according to his social context. Programs that promote the development of emotional intelligence levels among the adolescent population report the benefits of these interventions to moderate behavior, as well as to reduce its emotional and social consequences (Vega et al., 2022).

Therefore, having emotional skills has nowadays become a requirement for the success of subjects in all scenarios of their actions and a large part of their life cycle. Hence, it is necessary to recognize and strengthen these skills. As a result of the above, training spaces are considered as large scenarios that require inviting to rethink the development of their practices oriented not only to the development of contents but also to the execution of actions that contribute to an adequate approach of the tools related to being emotionally intelligent, being imperative for the academy and research environments to value the skills of being for their respective promotion and intervention.

## 5. Conclusion

This research enabled us to analyze the scientific production of ES and identify emerging lines of research. Even though several literary reviews have been conducted in the study of ES, there was no research presenting a bibliometric analysis that could show the networks and structures of the work of the main authors in this area.

Moreover, it is highlighted that the results were shown using a tree structure analogy, to explicitly guide the evolution of this knowledge area. The root’s research was reflected as the platform and starting point of this content, the trunk’s research provided the ES structure, and the leaves’ research is considered as clusters or subareas of research with regard to the subject, thus demonstrating three main subject matters—ES and education, personality, and ES and autism.

It is important to highlight that the explorations of this study have been developed mainly in countries of developed economies. However, in zones like Latin America, the research that aims to study this inquiry is in the initial phase. Nevertheless, the structural documents are important because they provide and set the grounds to continue with the methodology, improve models and design, and remain in time as important sources that value the learning of individuals’ ES.

ES has been necessary competency in the development and use of EI. Human beings that use this concept show greater advantages in generating positive responses and generate greater empathy to face problems and difficult situations. Because of the constant massive social problems that affect mental health, training is essential and we need to implement this in daily life to overcome obstacles, envision solutions, and make responsible decisions that provide tranquility and personal satisfaction.

This process allows us to perceive, as science provides us with more knowledge, the conceptualization of emotional education, personality development, and autism which will inevitably evolve in a more precise direction. The HE contributes to the development of emotional competence as a significant factor of effective and responsible citizenship, and its mastery favors a better adaptation to the context and tends to respond to life circumstances with greater chances of success.

Finally, in this study, important lines of research are observed, which are as follows:

- Qualitative research taking into consideration emotional expressiveness, understanding emotions, and regulating emotions and behavior.
- The impact of social skills training in solving social problems.
- Research on trends in the pedagogical training of social skills for professionals from different areas.
- Study of training assessment instruments in social and ES.
- Study of socio-emotional skills, personality traits, and constructions related to adopting structural equation models and plausible values.
- Impact of the development of pedagogical resources used by professionals and families in supporting children with ASD.

Although the purpose of this study was to develop a systematic bibliometric review of emotional skills, obtaining significant and useful results for the scientific and academic community, the search was focused on databases, such as WoS and Scopus, and was oriented at a general level on emotional skills. Therefore, for future bibliometric analyses, it is recommended to use more specialized databases and to limit the search or the number of research fields to certain areas of knowledge or disciplines to obtain more specialized results. In other words, for future work of this type, we suggest the inclusion of other thematic areas and the use of new search terms that allow the inclusion of other articles and a more detailed analysis of the metadata to investigate other current and future lines of research.

## Data availability statement

The original contributions presented in the study are included in the article/Supplementary material, further inquiries can be directed to the corresponding author.

## Author contributions

MM, DD, and MÁ contributed to the conception of the topic and bibliometric analysis, and wrote the first draft of the manuscript. MM, DD, IM, and JN-O wrote sections of the manuscript. JH-L, VB, JN-O, IM, and LC-T reviewed and edited the manuscript. MÁ, JH-L, VB, and LC-T drafted and revised the manuscript. VB and LC-T received funding acquisition. All authors contributed to the article and approved the submitted version.

## Funding

This study was supported by the project Psychosocial well-being of the academic community of the psychology programs of Norte de Santander, in relation to the experience of mandatory preventive isolation given by the coronavirus disease 2019 (COVID-19).

## Conflict of interest

The authors declare that the study was conducted in the absence of any commercial or financial relationships that could be construed as a potential conflict of interest.

## Publisher’s note

All claims expressed in this article are solely those of the authors and do not necessarily represent those of their affiliated organizations, or those of the publisher, the editors and the reviewers. Any product that may be evaluated in this article, or claim that may be made by its manufacturer, is not guaranteed or endorsed by the publisher.
