# Supplementary material for: A bibliometric analysis and literature review on emotional skills
Source: Front Psychol. 2023 May 24;14:1040110. doi: 10.3389/fpsyg.2023.1040110 (PMC10246769; doi:10.3389/fpsyg.2023.1040110)
Supplement: Supplementary file 1 [file Data_Sheet_1.docx]

Supplementary Material

# Tables

**Table 1** Search Parameters

| **Databases** | **WoS** | **Scopus** |
| --- | --- | --- |
| Consultation Period | 2000 - 2022 | |
| Consultation Date | 31/1/2022 | |
| Search Criteria | Title | |
| Type of Journal | All | |
| Search Terms | “emotional skill*” | |
| Results | 233 | 250 |
| General Results | 340 | |

**Table 2** Production per country

| **Country/Region** | **Number of publications** | | | |
| --- | --- | --- | --- | --- |
|  | **WoS** | **Scopus** | **Total** | **% of Total** |
| Usa | 53 | 44 | 55 | 16,18% |
| Spain | 32 | 20 | 38 | 11,18% |
| United Kingdom | 12 | 13 | 16 | 4,71% |
| Germany | 12 | 8 | 14 | 4,12% |
| Australia | 12 | 11 | 12 | 3,53% |
| Finland | 6 | 6 | 7 | 2,06% |
| Portugal | 6 | 6 | 7 | 2,06% |
| Italy | 5 | 6 | 6 | 1,76% |
| Turkey | 5 | 4 | 6 | 1,76% |
| Mexico | 4 | 2 | 6 | 1,76% |

**Table 3** Production per author

| **WoS** | | | | | **Scopus** | | |
| --- | --- | --- | --- | --- | --- | --- | --- |
| **Author** | **Total publications** | **Publications** | **Citations** | **H-index** | **Publications** | **Citations** | **H-index** |
| Fruyt, Filip K | 8 | 8 | 2631 | 48 | 8 | 8993 | 49 |
| John, Oliver P | 8 | 7 | 29289 | 62 | 8 | 32011 | 64 |
| Primi, Ricardo | 8 | 7 | 123 | 6 | 8 | 804 | 15 |
| Santos, Daniel D. | 8 | 7 | 77 | 4 | 8 | 128 | 5 |
| Takšić, Vladimir | 7 | 4 | 94 | 6 | 7 | 182 | 8 |
| Valencia Cobos, Jorge | 4 | 4 | 4 | 1 | - | - | - |
| Lechner, Clemens M. | 4 | 3 | - | - | 3 | 434 | 13 |
| Chamorro Miranda, Diana | 4 | 4 | 17 | 2 | - | - | - |
| Molander B | 4 | - | - | - | - | - | - |
| Schoeps, Konstanze | 4 | 3 | 116 | 7 | 3 | 131 | 7 |

**Table 4** Scientific Journals

| **Journal** | **WoS Database** | **Scopus Database** | **Total** | **% Total** | **Quartile** | **SJR (2020)** | **H-index (SJR)** |
| --- | --- | --- | --- | --- | --- | --- | --- |
| Frontiers in psychology | 8 | 8 | 8 | 2,35% | Q2 | 0,95 | 110 |
| Early child development and care | 7 | 7 | 7 | 2,06% | Q2 | 0,48 | 45 |
| Psiholoska obzorja | NA | 4 | 4 | 1,18% | Q4 | 0,16 | 3 |
| Pravention und gesundheitsforderung | NA | NA | 4 | 1,18% | Q4 | 0,2 | 11 |
| International journal of environmental research and public health | 3 | 3 | 3 | 0,88% | Q2 | 0,75 | 113 |
| Journal of applied developmental psychology | 3 | 3 | 3 | 0,88% | Q1 | 1,12 | 85 |
| Praxis & saber | 3 | NA | 3 | 0,88% | - | - | - |
| Interuniversity journal of teacher training-rifop | 3 | NA | 3 | 0,88% | - | - | - |
| European journal of developmental psychology | 2 | 2 | 3 | 0,88% | Q2 | 0,81 | 35 |
