# Supplementary material for: A bibliometric analysis and literature review on emotional skills
Source: Front Psychol. 2023 May 24;14:1040110. doi: 10.3389/fpsyg.2023.1040110 (PMC10246769; doi:10.3389/fpsyg.2023.1040110)
Supplement: Supplementary file 2 [file Data_Sheet_2.docx]

Supplementary Material

# Figures

**Figure 1.** Languages

**
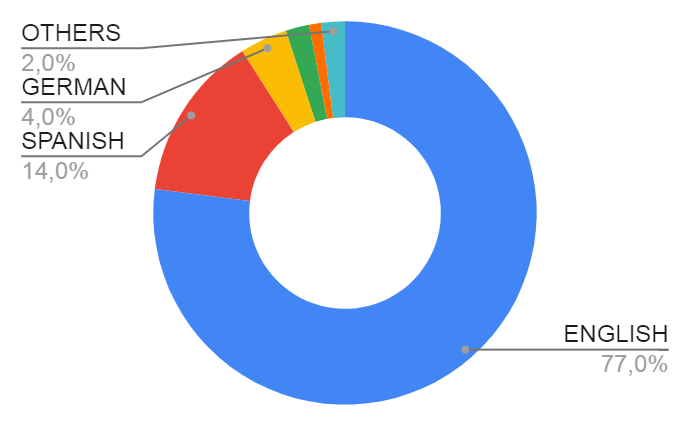
**

**Figure 2.** Scientific Production by years

**
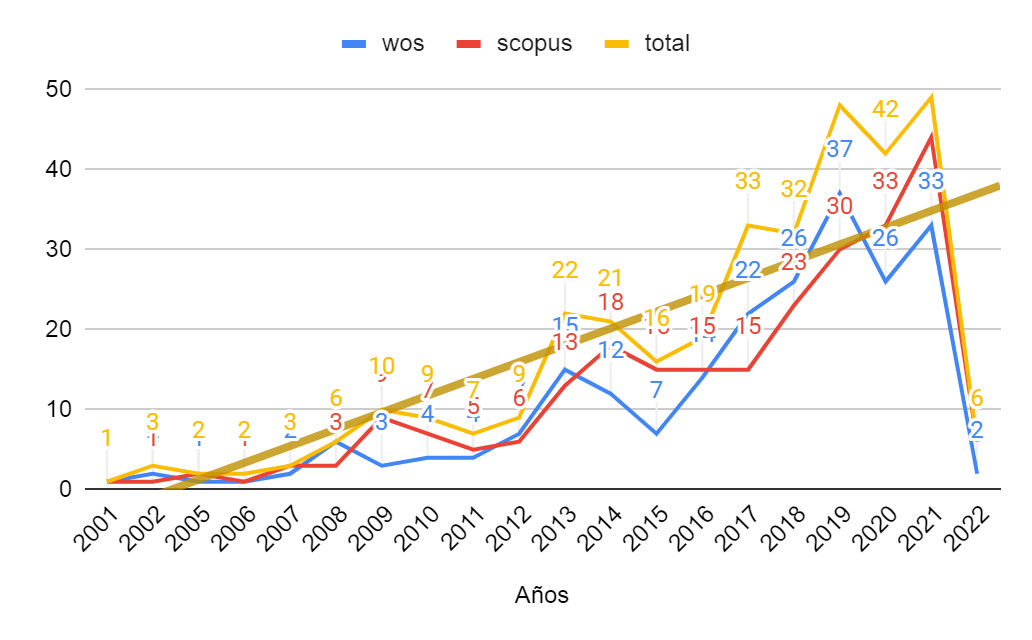
**

**Figure 3.** Networks

**Author co-citation network Collaboration network between authors**


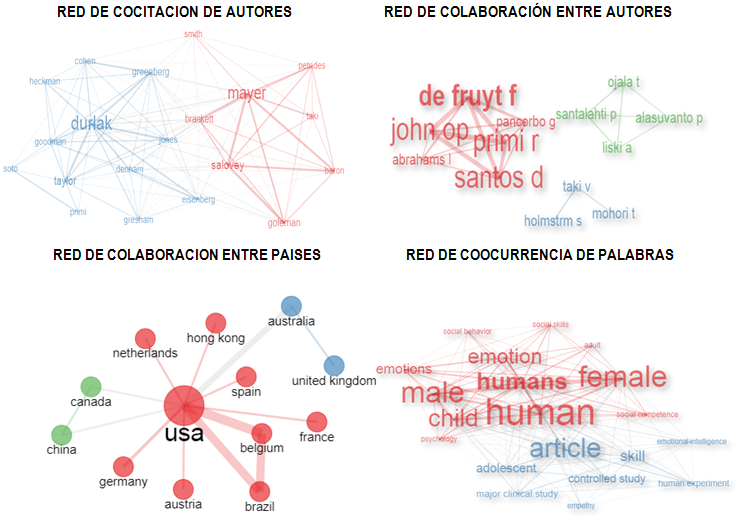


**Collaboration network between countries Word co-occurrence network**

**Figure 4.** Emotional Skills tree

Cluster 2: Emotional skills and personality


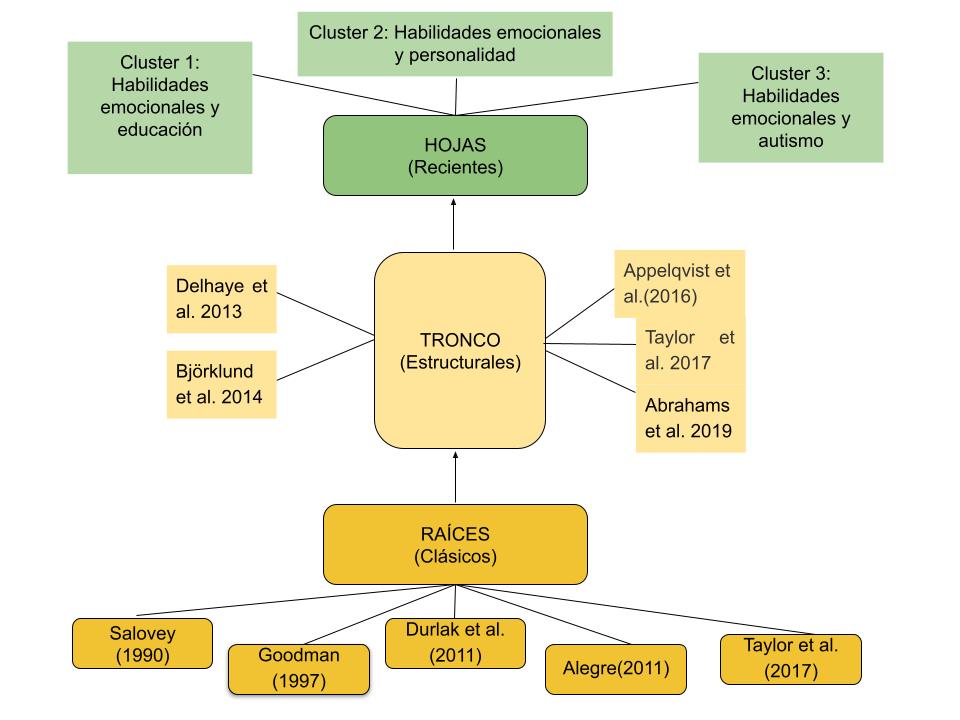


Cluster 3: Emotional skills and autism

**ROOTS (Classics)**

**TRUNK (Structural)**

Cluster 1: Emotional skills and education

**LEAVES (Recent)**

**Figure 5.** Perspective I


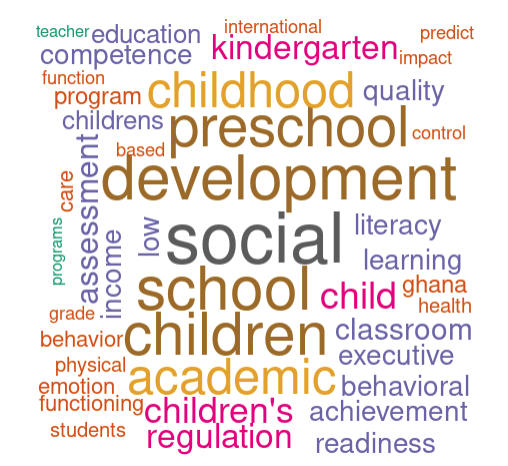


**Figure 6.** Perspective II


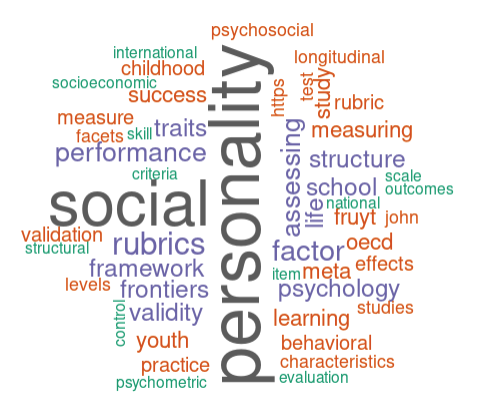


**Figure 7.** Perspective III

**
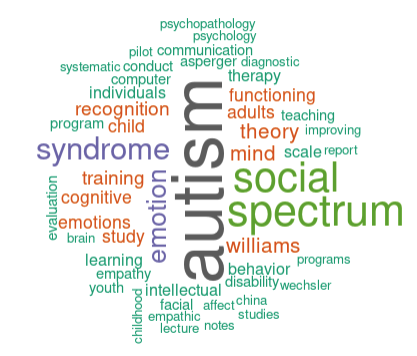
**
